# Exploring how interprofessional forces shape population-health competencies for clinical pharmacy trainees

**DOI:** 10.3389/fphar.2026.1865074

**Published:** 2026-06-12

**Authors:** Dixon Thomas, Muhammad Al-Shorbagy, Mahir Jallo, Sija Binoy, Danial Baker, Seeba Zachariah

**Affiliations:** 1 College of Pharmacy, Gulf Medical University, Ajman, United Arab Emirates; 2 College of Medicine, Gulf Medical University, Ajman, United Arab Emirates; 3 Internal Medicine, Thumbay University Hospital, Ajman, United Arab Emirates; 4 College of Nursing, Gulf Medical University, Ajman, United Arab Emirates; 5 College of Pharmacy and Pharmaceutical Sciences, Washington State University, Spokane, WA, United States

**Keywords:** clinical pharmacy training, force field analysis, interprofessional education, population health competencies, qualitative research

## Abstract

**Background:**

Interprofessional clinical training is increasingly recognized as essential for preparing pharmacy graduates to address both individual patient needs and broader population-health priorities. Yet how Doctor of Pharmacy (PharmD) students actually develop population-health competencies within clinical environments remains poorly understood.

**Methods:**

This qualitative research explored the learning frame with driving and restraining forces that shape population-health competence among final-year PharmD students during their clinical pharmacy training. Using semi-structured interviews were conducted with a clinical pharmacist, a medical doctor, a nurse, and three PharmD students at Thumbay University Hospital. ATLAS.ti software version 25 was used for data analysis.

**Results:**

Data was collected from three professionals and seven pharmacy students. Thematic analysis following Braun and Clarke’s six-phase approach generated 257 coded quotations and 17 themes. These themes were organized into three categories: learning frame themes, driving forces, and restraining forces. Findings show that students learn through iterative movement between population-to-patient and patient-to-population reasoning, supported by processes such as listening, observing, complementing team activities, validating clinical decisions, and implementing changes. Their development is shaped by driving forces including knowledge, compatibility, collaboration, integration, and trustworthiness, and restrained by limited knowledge, limited involvement, conflicting views, missing information, and unreliable performance. Together, these dynamics illustrate how interprofessional clinical environments can both foster and hinder the development of population-health competencies.

**Conclusion:**

This study highlights how population-health competence among PharmD trainees emerges through dynamic, interprofessional interactions that shape both their reasoning and clinical engagement. The interplay of driving and restraining forces within clinical environments reveals that supportive team processes can accelerate learning, while structural and relational barriers can impede it. Strengthening interprofessional integration and addressing these barriers may enhance the development of population-health competencies in future pharmacy training models.

## 1 Introduction

Healthcare systems are placing greater emphasis on collaborative practice, which involves coordinated efforts among healthcare providers from diverse backgrounds (Lüchinger et al., 2025; Geese and Schmitt, 2023). As a result, pharmacy education now goes beyond just medication expertise. It now includes competencies in communication, teamwork, systems thinking (the understanding of how different components of healthcare systems interact), and population health (which focuses on the health outcomes of groups of individuals) (Henman, 2020; Katoue and Schwinghammer, 2020; Van Eck et al., 2021). Clinical pharmacy training sites bring together pharmacists, physicians, nurses, and students. In these environments, learners encounter real-world examples of how population-level evidence, guidelines, and public health priorities shape patient care (Kobrai-Abkenar et al., 2024; Anderson et al., 2025; Nwizu et al., 2023; Ranchon et al., 2024; Moura Gomes et al., 2025). Despite this focus, there remains little clarity on how pharmacy students develop population health competencies in these interprofessional education (IPE) clinical settings (Ni et al., 2024; Mahmoud et al., 2025; Fahs et al., 2025).

Expectations for pharmacists in population health keep expanding. They now include roles in antimicrobial stewardship, chronic disease management, health literacy, and culturally responsive care (Sakeena et al., 2018; Dighriri et al., 2023; Taylor et al., 2025). Yet, most work placement training still focuses on patient-level tasks (Lea et al., 2025; Abu Farha et al., 2021; Nob et al., 2022; Peletidi et al., 2025). This leaves a gap in understanding how students link individual cases to broader population-based reasoning. Interprofessional settings add more complexity. Students must navigate diverse professional views, communication styles, and levels of involvement (Mmoloke et al., 2025; Crafford et al., 2025; Weber and Caward, 2025; Fahs et al., 2025; Alsharari et al., 2025). At the same time, they must integrate evidence, learn collaboration, and help make team-based decisions (Ascione, 2019; Aizawa et al., 2026; Bradley et al., 2021). Although experiential learning shapes professional identity and competence, little is known about the specific learning processes, supportive conditions, and barriers that affect how Doctor of Pharmacy (PharmD) students develop population health competencies during IPE clinical training (Grand-Guillaume-Perrenoud et al., 2025; Dong et al., 2025; Gauthier et al., 2025; Mukhalalati et al., 2024; Mukhalalati et al., 2022).

To address these gaps, this study explored how pharmacy students learn population-health competencies needed to improve the health outcomes of groups or communities in an interprofessional clinical pharmacy training environment. The aim was to identify driving and restraining forces, i.e., factors that facilitate or hinder learning in this context. The study is not restricted to IPE between students of different professions, but also include pharmacy student learning from pharmacy, nursing, and medical professionals. As the final year clinical training is a time of transition from education to independent practice, some notions are inclusive of pharmacy practice and pharmacists, not just students. The research question was: How do PharmD students develop population-health competencies in an interprofessional clinical pharmacy setting, and what driving (facilitating) and restraining (hindering) forces influence this process?

## 2 Methods

### 2.1 Study design

This study used a qualitative design recognizing that knowledge is co-constructed through participants’ and researchers’ experiences and interpretations. A phenomenological orientation guided the exploration of how individuals experience interprofessional clinical training and understand the learning processes that shape population-focused competence in PharmD students. The situated learning theory was used as the theoretical basis for this research (Mukhalalati et al., 2022). A force field analysis lens was applied to identify the driving and restraining forces influencing students’ development (Jayalakshmi et al., 2020).

### 2.2 Study setting and participants

The study was conducted at a tertiary-level care Hospital in the UAE, where PharmD students in their final academic year from a University in the UAE perform their clinical training. Purposive sampling recruited participants with direct experience in interprofessional clinical training reflecting on experiences of participants with regard to PharmD students. The reference was at times, the pharmacists or clinical pharmacist to touch upon what students aim to become. The sample included one clinical pharmacist, one medical doctor, one nurse, and a three final-year PharmD students. Thematic saturation was achieved by repeatedly collecting the data from the participants for deeper insights that adding new participants. This composition ensured key professions were represented in the interprofessional learning environment. All three professionals involved in the study had been involved with IPE student training at this practice site. All students had some exposure to IPE clinical training and completed 4–5 months of clinical training before being interviewed. Participants from different backgrounds and diverse experiences were intentional to enrich data collection. The perspective was of pharmacy students through their experience, pharmacy and interprofessional faculty who trained them. The force field analysis lens was primarily from a pharmacy profession viewpoint. The iterative coding process was expected to continue until thematic saturation was achieved.

### 2.3 Data collection

Data were collected through semi-structured interviews from January to March 2026. Data collection was planned until saturation of themes was achieved. The number of themes were restricted to core concepts that interconnects well to avoid crowding of the conceptual framework. PharmD students participated in one round of interviews, while the clinical pharmacist, nurse, and medical doctor participated in two rounds. The second round served as a member-checking process, allowing participants to validate initial coding, clarify interpretations, and provide additional insights. All interviews were audio-recorded using the Zoom platform, transcribed verbatim, and anonymized by omitting participants’ names from the results.

### 2.4 Data analysis

Thematic analysis used Braun and Clarke’s six-phase approach (Braun et al., 2019), supported by ATLAS.ti version 25. Coding was performed by two researchers at the study site and validated by other researchers. No artificial intelligence tools were used in the coding or thematic analysis. Analysis steps included data familiarization, generating initial codes, searching for themes, reviewing and refining themes, defining and naming themes, and producing the final thematic structure. The force field analysis lens organized themes into learning processes, driving forces, and restraining forces, reflecting dynamic influences shaping students’ competence development at both patient and population levels.

### 2.5 Researcher positionality

The research team operated from an insider–outsider positionality. Several researchers had pharmacy backgrounds, including the interviewer, providing insider insight into PharmD training and clinical pharmacy practice. To enrich interprofessional perspectives and reduce disciplinary bias, one researcher each from medicine and nursing contributed to study design, interpretation, and review of findings. Additional pharmacy researchers contributed to planning and iterative analysis. Reflexive discussions were maintained throughout the study to ensure that interpretations remained grounded in participants’ accounts rather than researchers’ assumptions. Additional faculty from other professions could enrich data, but for generating a unique framework with deeper insights from selected faculty with IPE invovlement was focused at this stage of the research. While the research is driven by the positionality and reflexivity of the researchers, one medical and one nursing professional and multiple students were added to contribute to the objectivity of the findings.

### 2.6 Trustworthiness and rigor

Multiple strategies were employed to enhance the credibility and dependability of the findings. Data triangulation was achieved by integrating interviews from students and healthcare providers. Investigator triangulation involved multiple researchers independently coding transcripts and discussing discrepancies. Peer debriefing sessions with qualitative experts provided external scrutiny of analytic decisions. An interview guide was maintained for the semi-structured interviews. Reflexive journaling documented the influence of the researcher’s assumptions and positionality. An audit trail of coding decisions, theme development, and analytic memos ensured transparency and methodological rigor.

### 2.7 Ethical considerations

Ethical approval was obtained from the Gulf Medical University Institutional Review Board (IRB-COP-FAC-136-Mar-2025). Oral consent was obtained from all participants. Participants’ real names were not included in the results. Participants were assured their care, training, or academic standing would not be affected by their decision to participate or decline.

## 3 Results

**FIGURE 1:**
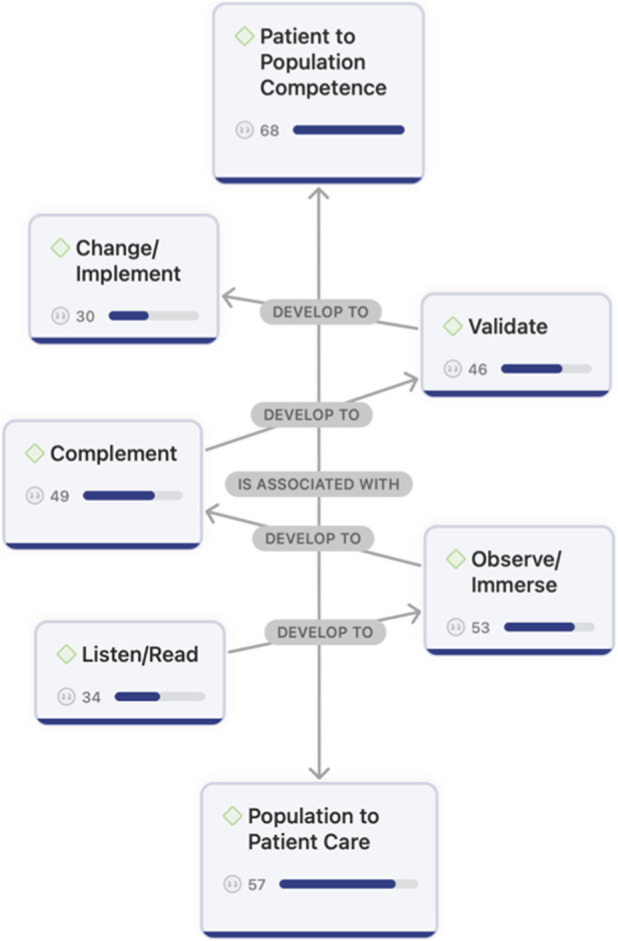
Clinical pharmacy learning frame of interprofessional population health competence.

**FIGURE 2:**
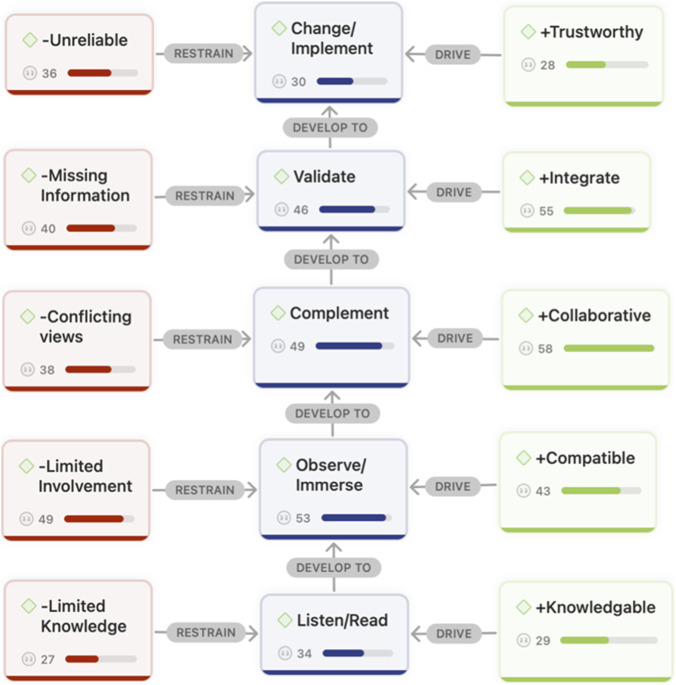
Driving and restraining forces in developing population-health competence.

Interviews with a clinical pharmacist, a nurse, a doctor, and seven final-year PharmD students yielded 257 quotations, which were synthesized into 17 themes. These themes were grouped into three overarching categories: (1) learning frame themes, (2) driving forces, and (3) restraining forces. They were shown in two conceptual frameworks of learning frame themes in Figure 1 and force-field analysis of driving and restraining forces in the Figure 2.

### 3.1 Learning frame themes

The learning frame describes how students move between population-level and patient care approaches. Students first use population-based evidence, such as guidelines and research, to inform individual patient care. With more clinical experience, they start to recognize broader population patterns and apply this reasoning in their work. Understanding of population feedback back to patient care. This back-and-forth process shows the dynamic nature of clinical pharmacy training.

Five learning themes emerged within this frame: Listen/Read, Observe/Immerse, Complement, Validate, and Change/Implement. These themes describe how students engage with patients and healthcare professionals in IPE clinical settings. Students listen to patients and team members, read clinical documentation and literature, and immerse themselves in the practice environment through observation and participation. They complement clinicians’ work by contributing to routine pharmacy activities, such as medication history-taking and discharge counseling. They validate prescriptions and medication processes to ensure safety and, at times, work with patients or professionals to modify care decisions. Some of these actions extend beyond individual encounters, influencing policies or system-level practices. Together, these learning activities support the development of both patient-level skills and population-based competence (Figure 1).

#### 3.1.1 Population to patient care

This theme reflects how learners and practitioners move from applying broad, population-level guidelines to making individualized patient-care decisions. It highlights the reliance on established protocols, the gradual development of clinical judgment grounded in evidence-based practice.

“There are specific guidelines and protocols; everyone agreed to follow that … So, where to look for information, and what information is required. So, instead of looking into everything and taking a long time, they would process everything faster.” (CP)

“I think all these details of the updated evidence-based medicine should be more refocused in the training of the pharmacist.” (M)

“Medication risk management for the patients about high-risk medications is there.” (St1)

“They just followed the guidelines and adjusted the dosage and this and that.” (St2)

#### 3.1.2 Patient to population competence

This theme describes how learners expand from individual patient encounters to understanding broader population-level needs. It encompasses recognizing patterns across cases, addressing the needs of special populations, providing culturally sensitive health education, and understanding how patient feedback can shape care processes and policies.

“Students are able to connect that whenever they get similar events … While learning theory, they are learning about special populations, so there is consideration for elderly patients with renal impairment, and pregnant populations.” (CP)

“Provide health education to the patients … Vulnerable population health literacy can be improved … The student needs to know what are the population-based competencies they are supposed to develop.” (N)

“We need some enhanced training curriculum and content, from the cultural competence, public health focus, and also continuous education … Product-centered approach, to patient-centered approach and community-oriented.” (M)

“They were going to add Galveston to the drug formulary, so we had to create a presentation on that, and it was interesting to learn about that.” (St2)

“I encountered one of the cases where we had to modify the treatment for the patient as she had just delivered 24 hours ago, and so we made sure that all medications were not passing through the breast milk.” (St3)

“The pharmacists that I’ve been with are not on any of the committees where they help persuade what is going to be on the formulary after a change in a guideline.” (St7)

#### 3.1.3 Listen/Read

This theme reflects how learners develop foundational understanding by actively listening to experienced practitioners and engaging with written information.

“When they are given a question, and they are asked to prepare about that. So they will read, connect the information.” (CP)

“Just to hear how the experienced team will approach the problem.” (M)

“I would go often with the clinical pharmacist to her stewardship meetings every so often and just listen in to other discussions with other professionals that speak about it.” (St2)

#### 3.1.4 Observe/Immerse

This theme captures how students learn by immersing themselves in real clinical environments and observing interprofessional interactions.

“They are observing. And seeing how people interact, they slowly develop that collaboration.” (CP)

“Giving more exposure in training will develop some competencies related to the population, population-based care … From beginning from admission till the follow-up, and even at home, what they are supposed to do, or what diet they are supposed to do.” (N)

“We’ve seen opioid and alcohol withdrawal management, and we saw how they conducted it with the people in the detox ward, and just saw how the pharmacist would interact with the physician and all that.” (St2)

#### 3.1.5 Complement

This theme highlights how interprofessional roles complement one another in patient care.

“There is an effort from everyone's side.” (CP)

“The pharmacist should have the same message like the doctor … They should also have one unique answer, like the physician prescribing the medication.” (M)

“Two pharmacists usually double-check to avoid any medication error or dosage error for each patient to avoid any error. So, there is double checking from two pharmacists to ensure that there is no medication error when administering high-risk medications to outpatients.” (St4)

“I did some cost-effectiveness analysis where it comes to the patient budget. For example, there’s two medications. The patient is not willing to pay that much, so we would give the other one. That’s what we did.” (St5)

#### 3.1.6 Validate

This theme describes the processes through which learners and practitioners verify information, ensure safety, and confirm alignment between patient understanding and clinical decisions.

“I ask the patient what the doctor told you about this … We also need to check the right drug, right dose, right duration, contraindications, whether there are drug-drug interactions.” (CP)

“Monitor even the patients who have taken the medication.” (N)

“We should have some feedback mechanism, both from the pharmacist and from the patient, to see how we … I mean, how we are going in the right way … Risk assessment for the patient who is able to fast Ramadan or not.” (M)

“They monitor them clearly, and they have their own specific guidelines that … it’s a hospital rule.” (St1)

“Go through the controlled medications to make sure that there’s none missing, like it’s aligning with the stock on their system. Stock recorded on their system aligns with what is on the shelf, to ensure that no prescription is missing, to ensure that stocks are appropriate.” (St4)

#### 3.1.7 Change/Implement

This theme reflects how learners and practitioners translate insights into action by modifying treatments, addressing lifestyle barriers, and implementing changes in care.

“Majority of the patients, they are happy whenever a student greets them and explains about the medications, asks about any concerns they have.” (CP)

“Overcoming some of the hindrances pertaining to lifestyle modifications.” (N)

“I encountered cases where we’d have to modify treatment.” (St3)

“She had to do a surgery, but they were prescribing for her aspirin, which was, like, in that case, should not have been prescribed. So I also told them about this overuse of this medication.” (St1)

#### 3.1.8 Driving and restraining forces

**FIGURE 3:**
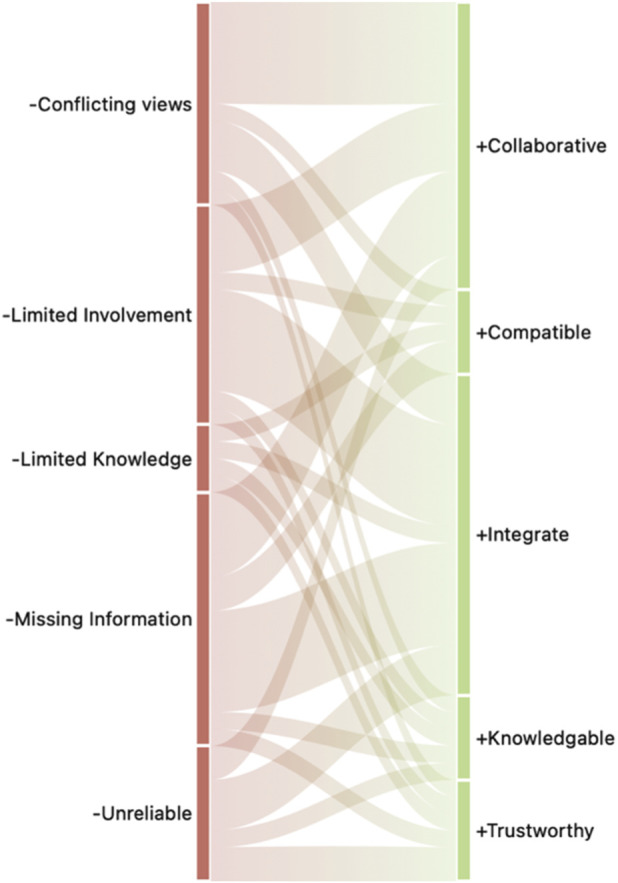
Sankey diagram of driving and restraining forces.

Five driving (in green color) and five restraining forces (in red color) were identified as connected with the learning frame (Figures 2, 3).

### 3.2 Driving forces

Five driving forces; Knowledgeable, Compatible, Collaborative, Integrate, and Trustworthy support students’ progression through the learning process (Figures 2, 3). Although each driving force aligns closely with particular learning activities, they operate across the continuum of patient and population care. Retaining essential knowledge, demonstrating compatibility and collaboration with patients and professionals, integrating information across sources, and making trustworthy recommendations all strengthen students’ ability to learn effectively and contribute meaningfully within interprofessional teams.

#### 3.2.1 Knowledgeable

This theme reflects how depth of knowledge, clinical recall, and pharmacotherapy understanding drive students’ ability to contribute meaningfully in interprofessional settings.

“Some students, especially, they have in-depth information, information that is connected, integrated. During the internship, pharmacotherapy knowledge also helps pharmacy students very well to collaborate with the medicine students. Often students say that what they learn in the rotation, they retain very well. They remember the incident clearly.” (CP)

“In the ICU, we had a case of a Kawasaki in a child, so the aspirin dose was a bit higher than what was recommended, so we had to say that.” (St2)

#### 3.2.2 Compatible

This theme highlights how compatibility, whether through attitude, willingness to learn, or cultural and linguistic alignment supports effective teamwork.

“Some students, naturally, they blend into the team. Some students who are not that knowledgeable, but they are ready, or they want to learn, or they want to learn from others, they go very well in any team. I won't be contradicting any of the … information that the doctor already gave.” (CP)

“When possible, the students speak the native language of the patient, assisting doctors who need translation, also for the nurses, who just sometimes give their translation, and I think all this will enrich the patient care … How to manage patients with low health literacy or diverse cultural needs across the healthcare system.” (M)

“Pictures to explain to people who may not understand, and they had different pamphlets in different languages, so that could help.” (St2)

“Follow the guidelines to reduce the chances of getting antimicrobial resistance.” (St1)

#### 3.2.3 Collaborative

This theme captures how collaboration across pharmacy, nursing, and medicine enhances patient care and strengthens student learning.

“Students progress in a good way in that collaboration with doctors and nurses … I have seen the collaborative work of pharmacy, nursing, and medicine students benefiting mutually.” (CP)

“We have the nursing person also, the medicine student also, and the pharmacy, who can talk simultaneously, and it will be a very effective discharge planning for giving the discharge summary to the patients.” (N)

“As part of escalating the dose, we write the note, but probably translating this to the patient, the pharmacist could be a very good aid for the multidisciplinary team.” (M)

“For inpatients, to be sent to the wards with labels saying that they are high-risk medications, just for all the healthcare professionals to take care before delivering the medication itself.” (St3)

#### 3.2.4 Integrate

This theme reflects how integration of knowledge, clinical processes, and interprofessional roles supports comprehensive patient care.

“Especially because in the university, they are learning different courses, which are integrated and connected.” (CP)

“From complete assessment, planning, care implementation, and also follow-up.” (N)

“Integrating them to analyze the improved care quality definitely may help to reduce the readmission of the patient, and also control the cost, especially the adherence issue for patients with chronic disease.” (M)

“Pharmacists would often check the lab values, like for example with lithium toxicity and with clonidine.” (St2)

“Sometimes some medications given in adult populations are not allowed to be given in children, due maybe to mechanism-wise or contraindications generally.” (St3)

#### 3.2.5 Trustworthy

This theme emphasizes how trustworthiness; built through competence, communication, and accountability, strengthens students’ roles within the healthcare team.

“Make it more appealing for the patient … Students would be really able to do good recommendations, identify problems.” (CP)

“Multidisciplinary team approach is very important to work with the doctor, with the nurses, with the other clinical pharmacists, because this will enrich their experience.” (M)

“I’ve noticed that one of the patients was receiving a medication although she was allergic to it, so then I reported it to the clinical pharmacist, and then she proceeded to reporting it to the physicians, and then they decided to stop, discontinue the medication.” (St3)

## 4 Restraining forces

Five restraining forces limit students’ development of patient- and population-level competence: Limited Knowledge, Limited Involvement, Conflicting Views, Missing Information, and Unreliable approaches (Figures 2, 3). These factors reflect gaps in understanding, restricted participation in clinical activities, differing perspectives among team members, incomplete information from patients or processes, and inconsistent performance under pressure. Together, these restraining forces illustrate the challenges students face as they navigate interprofessional clinical environments and attempt to build competence across both patient and population domains.

### 4.1 Limited knowledge

This theme reflects how gaps in foundational understanding, short-term exam-focused learning, and unfamiliarity with clinical information restrict students’ ability to fully engage in interprofessional practice.

“Many are studying for the exam, and they forget … they often say a few days after the exam itself, they forget what they learned.” (CP)

“Knowledge gap will also be there.” (N)

“I do not even know what the full acronym stands for.” (St6)

### 4.2 Limited involvement

This theme captures how restricted participation, hesitation, and limited exposure to key clinical activities hinder students’ development.

“Even students who have good knowledge, sometimes they are hesitant.” (CP)

“We need to develop that culture of interprofessional health literacy thing.” (N)

“Now we have the oncology department, which is now an expanding future prospective for the medication. I think pharmacy graduates should have an idea about this.” (M)

“Regarding the medication errors, I have not seen any.” (St5)

“Students are not necessarily involved with the committee … I’ve been involved with a lot of the antibiotics and seeing everything, and I never was involved with any of the antimicrobial committees. I do not think that’s something that students are heavily involved in.” (St7)

### 4.3 Conflicting views

This theme reflects how differing opinions, communication styles, and patient expectations can create tension and reduce willingness to collaborate.

“Conflicting views are possible … That makes people hesitating to collaborate. People talk in their own words. So, there could be different opinions … If patient is not trusting, not agreeing with the student, they will show that as a dislike.” (CP)

“There may be some administrative hindrances for interprofessional training.” (N)

“Unless the patients see the doctors, if you meet the pharmacist many times, this is not appreciated.” (M)

“I’ve seen, like, usually in wards, they tend to maybe over-prescribe or prescribe in certain situations some anti-inflammatory medication that’s not really necessary.” (St1)

“Some of the doctors were a little bit more hesitant to stop certain antibiotics.” (St6)

### 4.4 Missing information

This theme highlights how incomplete information, whether from students, patients, or clinical processes creates barriers to effective learning and care.

“Students usually think in one direction. When they see any problem, or when they suspect an issue, they are only connecting it to what MCQ question they have learned before. Connecting multiple information together, applying it for that patient's situation, that's challenging for the students who are starting the rotation.” (CP)

“Patient dispensed medication for two months, and then he comes to follow up after four months. What happened? He took medication from other side, he skipped the medication, because this will have an impact, especially for chronic disease and readmission.” (M)

“Definitely patients missed some information in the process.” (St6)

“I did not do the full summary of everything, but I helped look up information.” (St7)

### 4.5 Unreliable

This theme describes how inconsistency in student performance, pressure-driven responses, and variability among training pharmacists can undermine trust and reliability.

“It does not mean that all their recommendations would be correct … When they are under pressure, I have seen many students just talk something to escape from that pressure. So many of the information they are giving will not be accurate at that moment. They just want to escape that pressure.” (CP)

“Not all the pharmacists, training pharmacists, will be the same.” (M)

“A lot of times I feel like it can be also scary talking to doctors, especially if you do not have a good relationship with them or really know them.” (St6)

## 5 Discussion

The findings of this study illustrate how pharmacy students develop competence along a continuum that moves between population-informed patient care and patient-informed population thinking. Students learned by engaging in a set of interconnected activities, listening, observing, complementing, validating, and implementing changes within interprofessional environments shaped by clinical pharmacists, physicians, and nurses. This dynamic learning process reflects the iterative nature of experiential education, in which students continuously draw on population-level evidence to inform patient care and, in turn, use patient encounters to refine their understanding of population-based needs (Almoghirah et al., 2023).

The results align with existing qualitative research showing that IPE enhances students’ ability to collaborate and apply evidence-based knowledge in clinical settings. IPE strengthens students’ understanding of professional roles and improves patient safety through shared learning experiences (Kor et al., 2025). Similarly, practice-based IPE studies have shown that immersion in real clinical environments helps students integrate theoretical knowledge with practical decision-making and develop confidence in interprofessional communication (Kong et al., 2025). Experiential placements allowed pharmacy students to better understand how interprofessional teams function and how their contributions influence patient outcomes (Kennie-Kaulbach et al., 2023; Depasquale et al., 2025).

The driving forces identified, being knowledgeable, compatible, collaborative, integrative, and trustworthy, mirror characteristics described in broader IPE literature as essential for effective interprofessional practice. Reviews of pharmacy IPE consistently highlight foundational knowledge, communication skills, cultural competence, and trust as critical enablers of collaboration (Rawlinson et al., 2021; Balague-Viladrich et al., 2026; Lai et al., 2025). Positive attitudes, readiness to learn from others, and mutual respect underpin successful IPE experiences (Patel et al., 2025; Flood et al., 2022).

At the same time, the restraining forces limited knowledge, limited involvement, conflicting views, missing information, and unreliable performance reflect challenges commonly reported in experiential IPE research. Students often describe feeling underprepared or hesitant to participate fully in interprofessional discussions, particularly when hierarchies or unclear expectations are present. Students may struggle to assert themselves or contribute meaningfully when they lack confidence or when their roles are poorly defined (Alsharari et al., 2025; He et al., 2024; Boland et al., 2018). The present study similarly found that students’ involvement was sometimes restricted by their own hesitation or by clinicians’ reluctance to include them in decision-making processes. Conflicting views and missing information also echo findings from studies that show communication barriers, fragmented care processes, and varying levels of patient literacy hinder effective interprofessional collaboration (Rawlinson et al., 2021; Rojas-Ocaña et al., 2023; Ho et al., 2023).

The interplay between driving and restraining forces highlights the complexity of learning in interprofessional clinical environments. While supportive team cultures, strong knowledge foundations, and opportunities for meaningful participation can accelerate students’ development, gaps in knowledge, limited exposure, and hierarchical dynamics can impede progress. These findings reinforce the need for structured, intentional IPE design that ensures students are not only present in clinical environments but are actively engaged, supported, and empowered to contribute (Lindqvist et al., 2025; Donnelly et al., 2025).

Overall, this study contributes to the growing body of literature emphasizing the importance of experiential, IPE in preparing pharmacy students for population-focused practice. By illustrating how students navigate the continuum between population-to-patient care and patient-to-population competence, the findings underscore the value of clinical environments that foster both individual patient care skills and broader public health perspectives.

## 6 Limitations

This exploratory study was designed to understand what was happening within a specific interprofessional clinical pharmacy training site. The sample size was intentionally kept focused to capture the experiences of those directly involved. The primary perspective was from the pharmacy profession about competencies of pharmacy trainees, but for adding objectivity a medical and nursing professional was added to the interviewed. Data saturation was achieved with repeated data collected and coded. As with most qualitative inquiries, the findings reflect the perspectives of participants within one institutional context and may not represent all IPE environments. While interprofessional viewpoints were essential, it was also necessary to have a pharmacy predominance to develop training recommendations for pharmacy students. The study relied on self-reported experiences, which can be shaped by recall or social desirability. These limitations are typical for qualitative work of this nature and do not diminish the value of the insights gained, which help bring greater clarity to an area where guidance on training for population-health competencies remains limited.

## 7 Conclusion

The study concludes that pharmacy students develop competence through patient-care with population-health implications at clinical settings. Within IPE clinical settings, students learned by listening, observing, participating in team activities, validating clinical decisions, and implementing changes. These learning processes helped them translate guidelines and research into individualized care while also recognizing broader population patterns emerging from patient encounters. The results demonstrate that students’ development is shaped by the dynamic interplay between what they learn from population data and what they experience directly in patient care, reinforcing the idea that competence grows through repeated back-and-forth engagement across this continuum.

The study also highlights that students’ progression is influenced by a set of driving and restraining forces. Supportive forces such as strong foundational knowledge, compatibility with team dynamics, collaborative engagement, integrative thinking, and trustworthiness enabled students to participate meaningfully in interprofessional practice and to contribute to both patient-centered and population-focused decisions. At the same time, barriers such as limited knowledge, restricted involvement, conflicting views, missing information, and unreliable performance created challenges that slowed or disrupted learning. These findings emphasize that experiential learning environments can simultaneously foster and constrain the development of population-based competence, depending on the conditions students encounter. Further exploratory and confirmatory research shall be planned from the insights generated from this research.

## Data Availability

The raw data supporting the conclusions of this article will be made available by the authors, without undue reservation.
